# Estimation of dye concentration by using Kubelka–Munk and Allen–Goldfinger reflective models: comparing the performance

**DOI:** 10.1038/s41598-023-29264-x

**Published:** 2023-02-03

**Authors:** Mahdi Safi, Seyed Hossein Amirshahi

**Affiliations:** 1 Department of Color Physics, Institute for Color Science and Technology, Tehran, Iran; 2 Department of Textile Engineering, Textile Chemistry and Fiber Sciences, Amirkabir University of Technology, Tehran, Iran

**Keywords:** Theoretical chemistry, Chemistry, Engineering, Mathematics and computing

## Abstract

If the relationship between the reflectance function (K/S) and dye concentration (C) is known, the color of the dyed textile (R_∞_) and C could be predicted from each other. In the present work, the concentration value estimated from the reflectance data using two reflective models, i.e. the Kubelka–Munk and the Allen–Goldfinger is compared. First, the Allen–Goldfinger model was run by using the absorption coefficient of dyes in fiber, i.e. the unit k/s values instead of that in the solution. The results showed that the replacement of the unit k/s for the Beer–Lambert absorption coefficient in the Allen–Goldfinger model causes lower error in the prediction of the spectral reflectance factor as well as the dye concentration. However, this model did not lead to better results. Then, an inverse form was used to estimate the concentration of dyes from the corresponding spectral reflectance. Consequently, it was observed that the Kubelka–Munk model is still a more reliable method while benefiting from more simplicity than the Allen–Goldfinger model. The analysis of errors showed that the results deeply depend on different factors such as the applied concentration range as well as the dye spectral adsorption behavior.

## Introduction

One of the textile coloration techniques is dyeing which can be run during any stage in the manufacture of textile products taking different physical forms such as loose fiber, yarn, tow, top, woven, nonwoven, knitted substrates, or garments^1^. Studies have shown that nearly 5% of textile products must be re-dyed for several reasons. To achieve precise color for a dyed product with the desired depth and shade, it is necessary to carry out controls in different stages of dyeing to minimize dyeing mistakes^2^. Therefore, it is strongly recommended to do it in the initial stages of the process (dyeing bath) and the final stage (dyed product). The control in the dyeing process may be done in both discontinuous (offline) and online manners. Nowadays, several methods have been developed for predicting the dyeing behavior and its more precise control by monitoring the dyeing bath variables such as the used dye concentrations. These methods are based on the chemical and physical principles of dyeing and by analyzing the dyeing bath and absorption spectrophotometric data^3–8^. In all of them, an attempt has been made to determine the exact ratio and amount of dyeing bath components, especially the amount of dye or concentration. The importance of determining and controlling the concentration of dyes in the dyeing process is due to the following^5^:

- Dye, the most important chemical in the dyeing bath.
- Studying the behavior of dyestuffs under different dyeing conditions.
- Optimizing the dyeing process.
- Determination of the efficiency of the dyeing machine.
- Dyeing process control.

The dyeing process and process control in the dyeing of textiles can be found in detail in textbooks^9–11^ so that it is classically characterized based on UV–Visible spectroscopy analysis of the dye bath^12^. Usually, the well-known Beer–Lambert law shown in Eq. (1) is employed to determine the concentration of dye in solution and/or solid phase, i.e. fibers^13–16^.

1 $$ \log_{10} \left( {\frac{{\uptheta _{{\text{t}}} }}{{\uptheta _{0} }}} \right) = - \log_{10}\uptau _{{\text{i}}} =\upvarepsilon {\text{cb}} = {\text{A}} $$

where $\theta_{t}$ is the monochromatic radiant power transmitted by the absorbing medium, $\theta_{0}$ shows the monochromatic radiant power incident on the medium, $\tau_{i}$ is the internal transmittance which is equal to $\frac{{\theta_{t} }}{{\theta_{0} }}$, $\varepsilon$ and c demonstrate the molar absorption coefficient and the dye concentration, respectively, and finally, b and A respectively show the absorption path length and the absorbance. The Beer–Lambert law could be applied to a completely transparent medium in either gaseous, liquid, or solid forms. Difficulties may be experienced in solid materials, particularly when they show a degree of translucency. The problem is critical in fibrous materials, which show a great surface reflection and considerable shell scattering. In addition, analytical work such as extraction of dye from fiber and measuring the dye concentration in a solid phase is time-consuming and tedious work. Both dissolvings of fibers and extraction of dye are possible when the dyes in the fibers are aimed. Hence, it is interesting to develop an alternative method to determine the dye concentration, especially in the solid phase from easier methods^9,16^.

In recent years, the reflectance spectrophotometer which provides the spectral, as well as the colorimetric data of samples, become more available. Due to some types of difficulties, such as low scalability, the reflectance data have been rarely used for the analysis of a dyeing system^9,17^. Continuing interest in the modeling of color behaviors of industrial samples such as textiles, plastics, and papers led to the presentation of numerous theories such as Kubelka–Munk, Mie, Multilayer methods, Multiflux, Allen–Goldfinger, and other methods for instance Monte Carlo simulations, expert systems, neural networks. Generally, in all models, it was aimed to establish a reliable relation between the reflectance and the optical properties of the dye-fiber system^14,15,19–21^. The most important aspect in these theories is mapping between the spectral quantity, whether reflectance or its function and the amount of applied dyes. Berns introduces a term named "color model" for such relations^22^.

The problem of primary interest in quantitative analysis of a dye bath or a dyed textile sample is to determine the concentration with a minimum error. We, in the present work, studied the applicability two reflective models i.e. the Kubelka–Munk (K–M) and the Allen–Goldfinger, which is called the Geometry model, to prediction the dye concentration in fibrous material. The models are applied on a series of nylon 6 fabrics dyed with four acid dyes. The Geometry model was improved by using the absorption coefficient of dyes in fiber, i.e., the unit k/s values instead of the adsorption coefficients in the solution. Then, an inverse form was applied to estimate the concentration of dyes from the corresponding spectral reflectance by the implementation of the improved version of the Geometry model.

## Theoretical background

### Single-constant Kubelka–Munk: a model with a linear mapping

Undoubtedly, due to its simplicity, suitability for the description of practical problems, and ease of use, the Kubelka–Munk (K–M) theory is still the most popular model in the prediction of color in a variety of industries such as decorative and protective coatings, paints, paper, pigmented polymers, textiles, thermal insulation, biological systems, medical physics, and atmospheric physics. As published in 1931, the Kubelka–Munk single-constant/two-constant theories which play an essential role in color science and technology, are a two-diffuse light fluxes model, with one flux proceeding "downward", and the other simultaneously "upward". It is a fundamental approach to modeling the appearance of turbid materials. The total reflectance from a coating surface which includes (a) the surface reflectance of the coating surface; (b) the internal remission of the coating; and (c) the surface remission from the substrate, can be related to the coating's absorption and scattering coefficients using Kubelka–Munk theory. Unlike the two-constant theory, the single-constant theory has been recommended to analyze the behavior of systems such as a dyed fiber in which the scattering amount of substrate is large in comparison with the total scatter provided by the dyes. The model’s mathematics can be found in detail in many publications^13,14,23–30^.

According to the single-constant K–M theory, a non-linear function of reflectance of an opaque substrate is proportional to dye concentration, if the surface reflectance is neglected. The K–M equation links a function of reflectance of a dyed substrate to the amount of dyestuff present in the fiber, as shown in Eq. (2):

2 $$ \left( {\frac{{\text{K}}}{{\text{S}}}} \right)_{\uplambda } = \frac{{\left( {1 - {\text{R}}_{{\infty .\uplambda }} } \right)^{2} }}{{2 \times {\text{R}}_{{\infty .\uplambda }} }} = \left( {\frac{{\text{K}}}{{\text{S}}}} \right)_{{{\text{sub}}.\uplambda }} + \sum {\text{C}}_{{\text{i}}} \upalpha _{{{\text{i}}.\uplambda }} $$

where $\left( {\frac{{\text{K}}}{{\text{S}}}} \right)_{{}}$ and $\left( {\frac{{\text{K}}}{{\text{S}}}} \right)_{{{\text{sub}}.}}$ show the K–M function of dyed and mock-dyed substrates. ${\text{R}}_{{\infty .\uplambda }} { }$ gives the reflectance of monochromatic incident light from an opaque material.$\alpha_{i,\lambda }$ is the unit k/s value of dye (slop of K/S versus concentration in the linear region) for a monochromatic radiant power on a given substrate and C shows the dye concentration in the substrate. K and S indicate the coefficients of absorption and scattering of monochromatic light of an opaque substrate. Equation (2) shows a linear relationship between the dye concentration and the reflectance function of the opaque media. It is supposed that the reflectance function is a linear function of dye concentration and the relation is valid up to a certain amount of concentration. Simply, it means the dye concentration could be determined by accessing the reflectance factor of dyed fiber (at least for the low to medium concentration ranges) if the value of $\alpha$ would be available^31,32^. An applicable method was introduced by Safi et al*.* to determine and extend the range of the scalability of the K-M model as well^33^. Despite the vast applications of the model, studies proved some inconsistencies between the predictions of the model and those obtain practically. This was due to some assumptions that were introduced to simplify the model. Consequently, the model is not able to explain and estimate the effects of some events such as wetting^34–38^, non-homogenous distribution of dye concentration (ring dyeing)^35,40^, the thickness of fiber^34,39^, surface texture (nap)^34^, the roughness of a surface^40,44^, the polarization of incident light^21,35,41^ on the color.

To overcome some of the limitations of the K-M model, other optical models have been developed for the analysis of the reflective behavior of colored objects. One of them, which is relatively easy in comparison with other models, is the Geometry model^15,42^.

### Geometry: a model with a non-linear mapping

The Geometry model was developed by Allen and Goldfinger in 1972 for predicting the color of textile fabrics. The model allows the determination of the optical behavior of an array of filaments, representing a textile fabric, such as reflectance, absorbance, and transmittance, from the individual variables of the fiber-dye system, i.e. the ratio of the refractive index of the fiber to that of the surrounding medium, the absorption coefficient of the dye, the filament diameter, as well as the applied dye concentration. In the Geometry model, the independent values of the dye concentration, C, and the dye absorption coefficient in the filament, k, are not known, while their product, Ck, is available. Once again, the use of k here should not be mixed up with the absorption coefficient value, K, of the K-M model.

**Figure 1:**
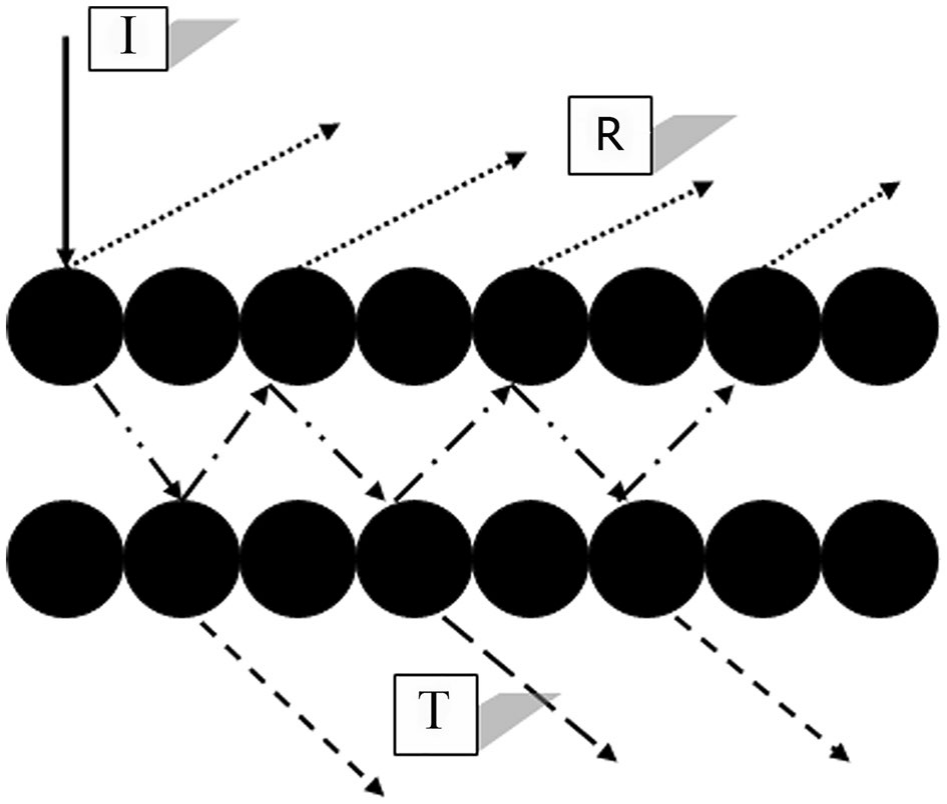
A graphic representation of incident light modification on the first layer of the filament array^35^.

A brief description of the model for implementation in a textile fabric could be stepped as follows^34,40^:

- The textile in form of fabric is represented by infinitely wide parallel layers of individual, isotropic, cylindrical, and identical filaments.
- All non-dyed filaments are completely transparent.
- The diameter of the filaments is the same over the medium and much larger than the wavelength of incident light.
- In the simplest form, the distribution of applied dye concentration throughout the filament is uniform.
- The incident parallel beam of light normally falls on the first layer and is modified as follows: a fraction is reflected from the surface $\left( s \right)$, a fraction is transmitted into the layer in the same direction of incidence $\left( t \right)$ and a fraction is absorbed by the dye $\left( a \right)$ [Eq. (3) and Fig. 1], so that:
  3 $$ {\text{I}} = {\text{I}}_{{\text{o}}} = {\text{t}} + {\text{s}} + {\text{a}} $$
  where $I_{ \circ }$ is the unit of incident intensity. The intensity of light transmitted and reflected from the first pair of layers is given by Eqs. (4) and (5), respectively.
  4 $$ {\text{T}}_{1} = {\text{E}}^{2} \left( {1 - {\text{a}}} \right)^{2} /\left[ {\left( {1 + {\text{E}}} \right)^{2} - \left( {1 - {\text{a}}} \right)^{2} } \right] $$
  5 $$ {\text{R}}_{1} = \left( {1 - {\text{a}}} \right)\left( {1 + {\text{T}}_{1} } \right)/\left( {1 + {\text{E}}} \right) $$
  where E is the ratio of transmitted light to reflected light (t/s) and a is calculated from the fraction of transmitted light for a single pass through the filament using the Beer–Lambert law.

The total amount of transmitted and reflected light for the complete filament array can be estimated by recursive Eqs. (6) and (7).

6 $$ {\text{T}}_{{\text{N}}} = {\text{T}}_{{{\text{N}} - 1}}^{2} \left( {1 - {\text{R}}_{{{\text{N}} - 1}}^{2} } \right) $$

7 $$ {\text{R}}_{{\text{N}}} = {\text{R}}_{{{\text{N}} - 1}} \left( {1 + {\text{T}}_{{\text{N}}} } \right) $$

where the subscript $N$ is the 2nth layer of the filament array. To predict the reflectance of fabric, $T_{N}$ should be adjusted to a minimum close to zero as shown in Eq. (8)^18,34^.

8 $$ {\text{T}}_{{\text{N}}} \le \left( {10^{ - 4} - 10^{ - 6} } \right) \cdot {\text{R}}_{{\text{N}}} $$

The mathematical equations for reflectance factor prediction by the Allen–Goldfinger model are too lengthy for a full description. Full details, such as the propagation of incident light in a fiber cross-section, could be found in Reference 35. The assumptions have been recommended to apply the Allen–Goldfinger model to predict the behavior of a dye-textile system as follows:

- The light beam hits the first plate or layer of material perpendicularly (as a bunch of parallel rays) and then diffuses to the next layers.
- The fibers are so close to each other that all the irradiated light hits the surface of the fiber, but the refraction and reflection that occurs as a result of light hitting one fiber is not affected by the adjacent fiber.
- Fibers are geometrically assumed to be cylinders with a circular cross-section and a diameter equal to and greater than the wavelength of the light emitted.
- The fibers are assumed to be isotropic and without any diffusion, and an average refraction coefficient is considered for them^35^.
- The distribution of the amount of dye is uniform in all the fibers, so the amount of transmission and reflection of each layer is considered constant and equal.

Despite the complexity, the Geometry model can be extended to describe some practical issues in textile processing such as follows^21^:

- Lack of access to deep shade for micro-fibers requiring extra consumption of dye.
- Effect of different media on the appearance of the fabric.
- Effect of physical finishing methods such as calendaring on appearance, etc.

In addition, some modifications have also been made to this model to improve its performance of this model for textile applications. For example:

- Non-cylindrical fibers^38,43^
- Fibers containing specific delustrants^26^
- Colorant mixtures^44^
- Fibers with skin–core structure^45^
- Optically anisotropic fibers^41^
- Ring-dyed filaments and yarn^40^
- Prediction of the dry color appearance from a wet state^20,46^
- Describing radiative properties of Nano pigment Coated Fabrics^47,48^
- Modeling the dye fading behavior^49^

## Experimental

### Materials

Green 20, Yellow 25, Red 128, and Green 25 were employed. Fabrics were knitted from nylon 6 filament yarn (68 filament bundle of 200 denier yarn) free from delustring agents. The filaments benefited from circular cross sections with a radius of 10 microns and the reflective index of the polymer was 1.55 and was manufactured by a domestic supplier named Alyaf Company. Other used chemicals were of analytical grade.

### Methods

Samples were dyed with classical dyeing. The fabrics were scoured before dyeing by using 2 g/l of nonionic detergent at 60 °C for 30 min. The samples were then washed in cold distilled water and dried in air. Finally, the fixation of samples was carried out with boiling water for 30 min before drying and dyeing. The dyeings were carried out in a laboratory-type dyeing machine (Linitest) at 40:1 liquor to good ratio in a buffer containing 0.5 ml/l acetic acid and 2.5 g/l sodium acetate to keep the solution pH = 5. The final temperature of dyeing was 98 °C. Dye concentrations in terms of percentages on the weight of fiber (% o.w.f.) were 0, 0.05, 0.1, 0.2, 0.3, 0.4, 0.5, 0.6, 0.7, 0.8, 0.9, 1.0, 1.2, 1.4, 1.6, 1.8, 2, 2.5, 3, 4, 5 and 6%. Finally, the dyed samples were rinsed.

### Measurements

The spectral absorption of all solutions was measured by Cary 100 Scan UV–Visible spectrophotometer from Varian. The spectral reflectance factors of dyed specimens were measured with a Color-Eye 7000A spectrophotometer from GretagMacbeth from 400 to 700 nm at 10 nm intervals with the specular component excluded.

### Computation of geometrical parameters and the error

In the Geometry model, the spectral reflectance factor of dyed fabric is directly correlated to its geometry and optical properties as well as the product of the dye concentration (C) and the adsorption coefficient of dye in the filament [Eq. (9)].

9 $$ {\text{R}} = {\text{f}}\left( {{\text{Ck}}.{\text{m}}.{\text{D}}} \right) $$

The reflectance factor (R) is a function of the product of dye concentration and the adsorption coefficient of the dye in the filament (Ck), the ratio of the refractive index of the filament to the surrounding medium (m), and the diameter of the filament (D). The value of m and D in $\mu m$ are accessible. In the Geometry model, it is assumed that independent values of C in g dye/100 g and k as lit/mg-cm are not known. Therefore, their product as Ck in 1/$\mu m$ is used. It is obvious that at low concentrations, the dye bath is completely exhausted. Consequently, as an approximate value, the % o.w.f. value was used instead of C. However, similar to the K-M model and at high concentrations, this replacement will cause a deviation in concentration estimation. In addition, the calculations were performed by using both the unit k/s values and the Beer–Lambert absorption coefficients as a measurement of k. The curve’s slope in the linear region of K/S against the concentration (C) was considered as the absorption coefficient of the dye in the fiber (unit k/s). To estimate the concentration with the Geometry model, a reverse method was used by a written program in MATLAB R2015a. In the other words, after running the correlation shown in Eq. (9), for an unknown sample with a given reflectance value, a range of concentrations was introduced into the program as inputs. At each step, the acquired error of the predicted reflectance factor relative to the real reflectance factor was calculated. The point with zero or a minimum error was determined as the corrected concentration. To evaluate the errors, the Error % function was used according to Eq. (10)^33^.

10 $$ {\text{Error\% }} = \left( {\frac{{\left[ {\sum \left( {{\text{X}}_{{{\text{Calculated}}}} - {\text{X}}_{{{\text{Measured}}}} } \right)^{2} /{\text{n}}} \right]^{0.5} }}{{{\overline{\text{X}}}_{{{\text{Calculated}}}} }}} \right) \times 100 $$

where ${\text{X}}_{{{\text{Calculated}}}}$ and ${\text{X}}_{{{\text{Measured}}}}$ represent the calculated variable from the model and the measured variable. n is the data point number. In estimating the concentration, the ${\text{X}}_{{{\text{Calculated}}}}$ ${\text{X}}_{{{\text{Measured}}}}$ could be replaced for the concentration values estimated from the model and the actual ones, respectively. Equation (10) shows a relative error that simultaneously considers the effects of the mean value and the number of samples or measured points in calculating the total error value. This results in a better and more accurate estimate of the amount of error and the accuracy of the concentration estimation operation. For the error analysis of a spectral property such as a spectral reflectance that predicted by the Geometry model, n indicates the number of wavelengths for which calculations were done. As mentioned above in Eq. (10), the error values are normalized and related to the number of data points and the mean values^46^. From the statistics point of view, high accuracy and reasonable certainty are acquired with the values of error $\le$ 5%^18,48^.

## Results and discussion

### Prediction of reflectance factor by the Geometry model

Even though the Geometry model requires some specific parameters of the textile material, the actual properties of a textile fabric in no way resemble the filament array of the model. It seems that the incompatibility of physical properties would be a key point in the accurate estimation of dye concentration.

**Figure 2:**
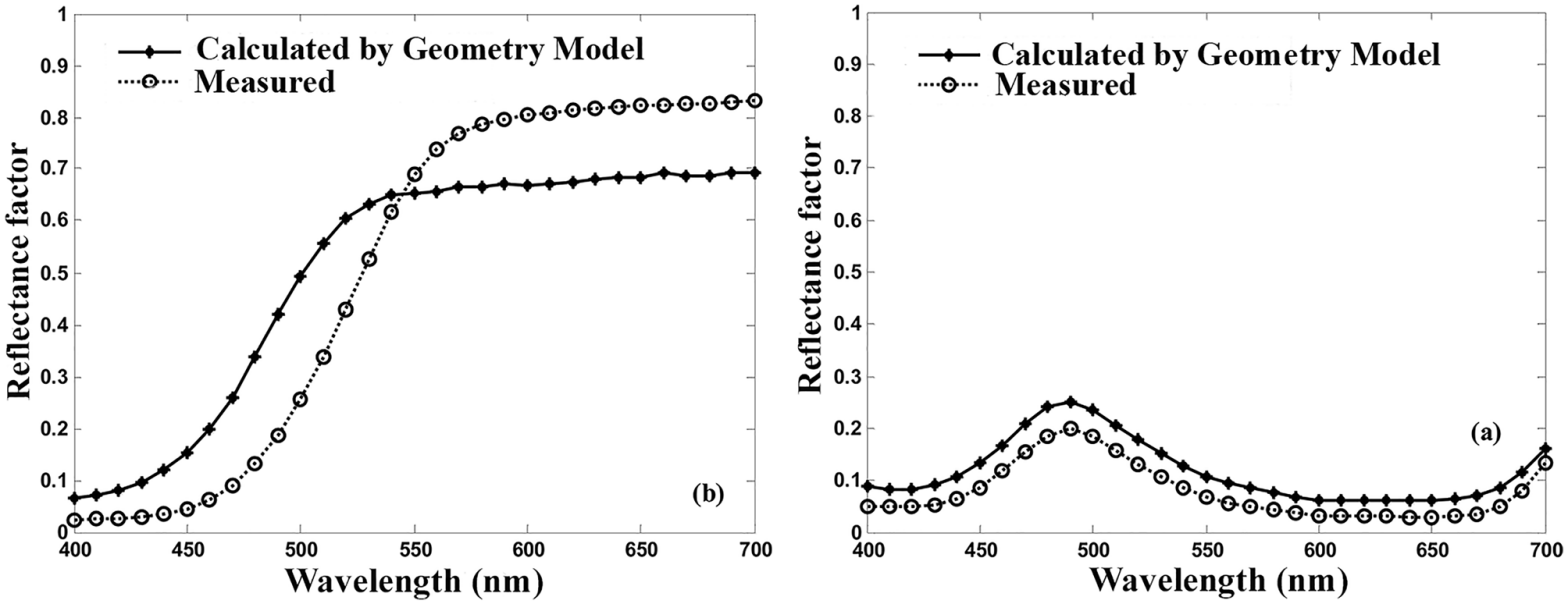
Estimated and actual spectral reflectance by the Geometry model for Acid Yellow 25 (left) and Acid Green 25 (right) at 1% o.w.f.

To run the program, first, the requirements of Eq. (9) should be defined. The Beer–Lambert coefficient was assigned to parameter k in the model. Figure 2 shows the spectral reflectance factor calculated by the Geometry model in comparison with those measured for two fabrics dyed with Acid Green 25 and Yellow 25 at 1%. Despite similar trends, the numerical values obtained from the model are greater than the actual values. The same results were observed for the samples dyed with other dyes over the applied concentration range. Furthermore, there is a noticeable inconsistency between the calculated and the actual reflectance at long wavelengths of the spectrum for yellows. It seems that high reflectivity over most regions of the visible spectrum for the yellows led to some types of errors in calculating the Beer–Lambert coefficients. Consequently, the estimated reflectance is not similar to the actual values at those wavelengths.

It seems, with a gradual increase in the concentration, the scalability of the reflectance data decreases. The reason relates to the deviation of reflectance spectra from linearity due to the limitations in the refractive models, as well as the non-linear dye uptake by the fiber. Such behaviors can be affected by various parameters including the region of concentration, the type of medium (aqueous or fibrous), and the wavelength^33^. Hence, the performance of the model in different ranges of concentrations was aimed and, the applied concentration range was divided into three categories, i.e. low (0.0–0.6%), medium (0.7–2.0%), and high (2.5–6.0%). Table 1 shows the analysis of the Geometry model performance in predicting the spectral reflectance in different ranges. In each part, the error of the prediction of reflectance is reported as the mean value.

**Table 1:** The mean error of the predicted reflectance factor by putting the Beer–Lambert coefficient into the Geometry model.

Dye	Mean error (%)		
	Low concentrations (0.0–0.6%)	Medium concentrations (0.7–2.0%)	High concentrations (2.5–6.0%)
Acid Green 25	37.93	63.12	93.67
Acid Yellow 25	21.61	32.38	56.98
Acid Red 128	41.34	62.96	70.73
Acid Green 20	191.75	211.55	111.04

**Figure 3:**
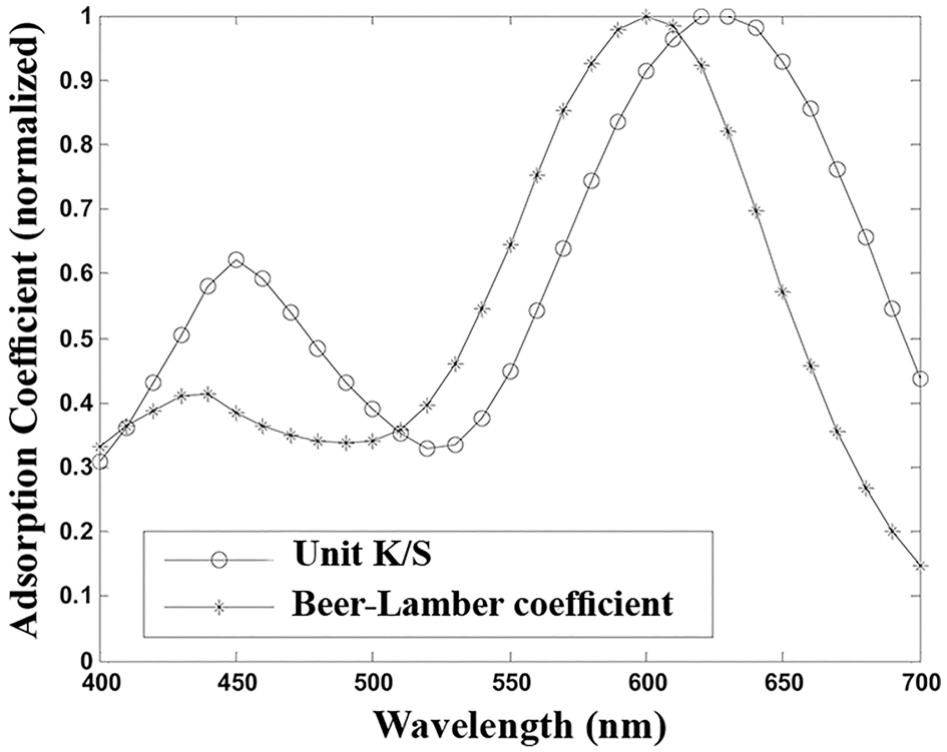
The normalized spectral behavior of the Beer–Lambert absorption coefficient and unit k/s for Acid Green 20.

The large values of error in Table 1 show that the Geometry model prediction was generally poor. Furthermore, the acquired errors in the different concentration ranges are significantly different. The prediction calculated error value for Acid Green 20 is greater than those for the other dyes. The employed green benefits from very low scalability that originates from the high color strength. The evaluations showed that the spectral reflectance function K/S of this dye begins to deviate from linearity in low concentrations i.e. after 0.3% o.w.f. Therefore, the error mean as indicated in Table 1, quickly increases. However, the predicted error at the concentration of 0.3% o.w.f. was calculated as 5.52%. It is no surprise that the errors are large since the Beer–Lambert coefficient is not likely to be valid in the filaments. Theoretically, the Beer–Lambert coefficient and the unit k/s from a series of dyeings prepared at low concentrations (calibration dyeings), should convey the same meaning. Both indicate the same property of the dye but in two different media, i.e., aqueous and fibrous (solid) states. In Fig. 3, the spectral behavior of the Beer–Lambert coefficient and the unit k/s for Acid Green 20 are compared. The Beer–Lambert coefficient has a dimensional property of conc^−1^ distance^−1^ whereas the unit K/S value is expressed in the form of conc^−1^. Hence, in Fig. 3, the normalized forms are plotted.

**Figure 4:**
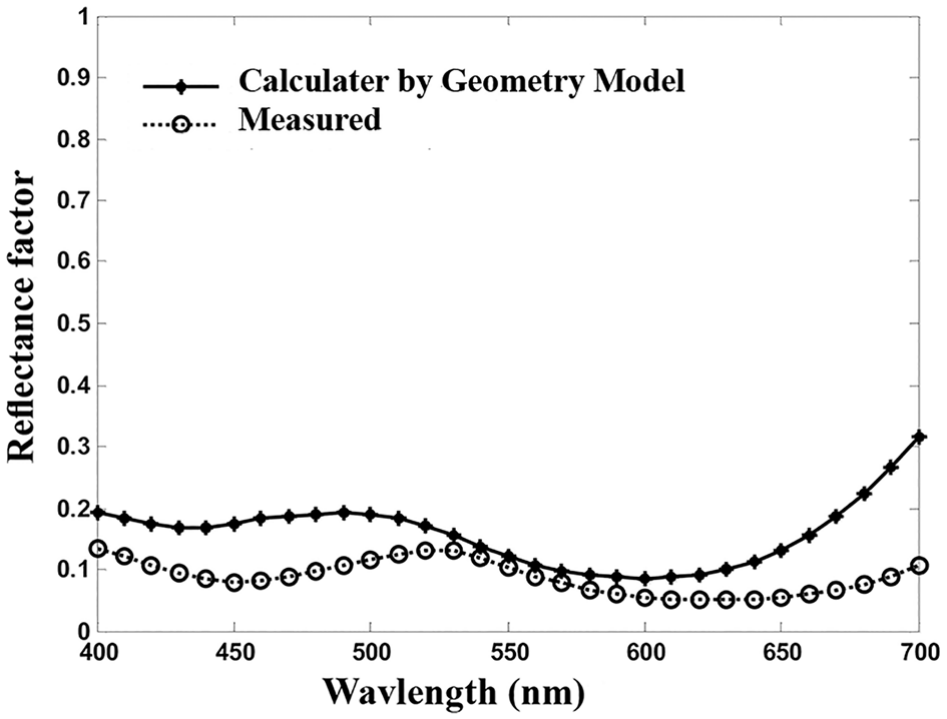
The actual and predicted spectral reflectance factors by using the Beer–Lambert coefficient in the Geometry model for Acid Green 20 at 0.2% concentration.

Figure 3 shows that the two coefficients are not necessarily related. Consequently, the values of the Beer–Lambert absorption coefficient are smaller than those of the unit k/s values in most wavelengths. This confirms the results shown in Fig. 2, that the calculated reflectance factors were more than those measured. The contrast backs to the interaction of the dye and fiber that in turn gives a specific spectral shift, especially at the wavelength of maximum adsorption^18^. The interaction would affect the prediction of the spectral reflectance by the Geometry model. To clarify the subject, the actual and estimated spectral reflectance factors by the Geometry model are plotted in Fig. 4, using the Beer–Lambert coefficient for Acid Green 20 at 0.2% concentration.

**Figure 5:**
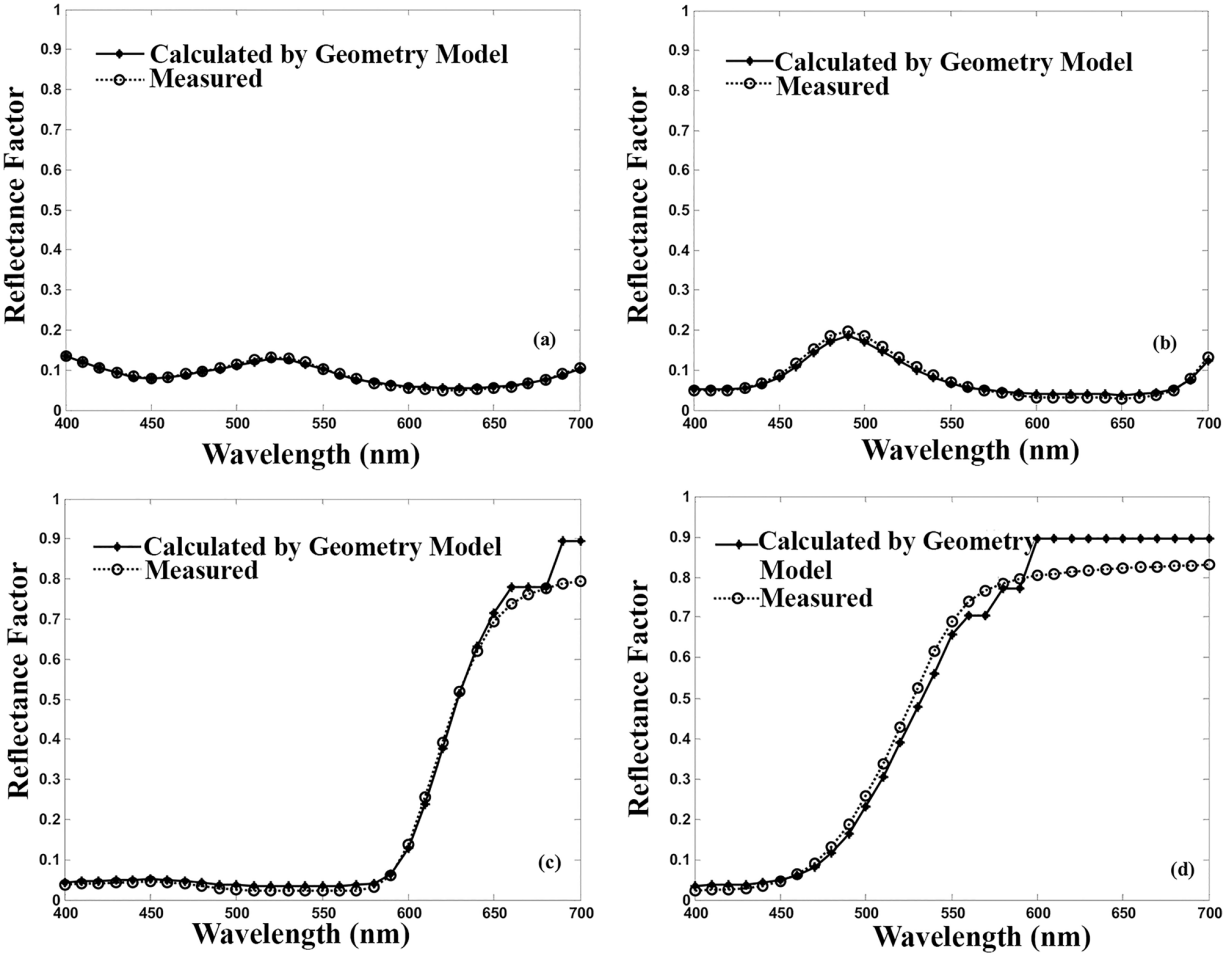
The actual and estimated spectral reflectance factor by Geometry model using the unit k/s. (**a**) Acid Green 20 at 0.2%, (**b**) Acid Green 25 at 1.0%, (**c**) Acid Red 128 at 0.9%, (**d**) Acid Yellow 25 at 1.0%.

Figure 5 illustrates comparing the actual as well as the predicted spectral reflectance factors for the selected dyes computed from the Geometry model while the unit k/s values are used. Apart from the area with very high reflectivity properties, that is to say, 600–700 nm for yellow and red samples, the estimated spectral reflectance factor by the Geometry model approximately matches the actual ones. The resulting error values are presented in Table 2.

**Table 2:** The mean of error of the predicted reflectance factor by employing the unit k/s in the Geometry model.

Dye	Mean error (%)		
	Low concentrations (0.0–0.6%)	Medium concentrations (0.7–2.0%)	High concentrations (2.5–6.0%)
Acid Green 25	8.45	14.13	21.41
Acid Yellow 25	11.57	20.11	28.33
Acid Red 128	18.06	71.81	86.77
Acid Green 20	8.45	14.13	21.41

The results in Table 2 show that at low concentration ranges, a considerable improvement could be observed. However, the mean errors at medium to high concentrations are still problematic.

### Concentration estimation

According to Eqs. (2) and (3), if the relationship between K/S and the dye concentration C were known, the reflectance value of the dyed textile (R_∞_) and C could be predicted from each other. Equation (3) is a basis in color-matching in computer-aided color matching [54].

11 $$ {\text{c}} = \frac{{\left( {\frac{{\text{K}}}{{\text{S}}}} \right)_{\uplambda } - \left( {\frac{{\text{K}}}{{\text{S}}}} \right)_{{{\text{sub}}.\uplambda }} }}{{\left( {\frac{{\text{K}}}{{\text{S}}}} \right)_{{{\text{unit}}.\uplambda }} }} $$

**Figure 6:**
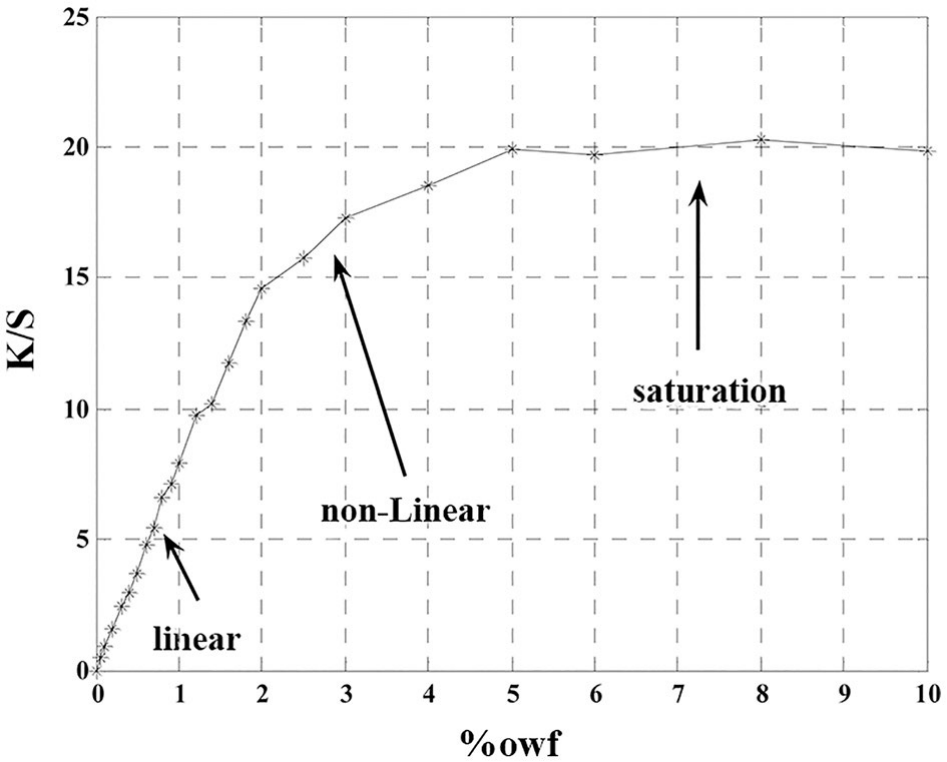
Different zones benefit from different adsorption behaviors of Acid Red 128 versus concentration at λ_max_.

However, the perfect proportionality of $\left( {\frac{{\text{K}}}{{\text{S}}}} \right)_{{{\text{unit}}.\uplambda }}$ and the concentration, c does not occur in practical dyeing, and deviations from linearity could happen. Therefore, as demonstrated in Fig. 6, the adsorption behavior of a dye on textile material would be described into three zones, linear, non-linear, and saturated stages^33^.

Conventionally, the unit k/s value which is determined at the linear zone and the wavelength of maximum absorption is commonly used for estimating the concentration. To estimate the concentration with the Kubelka–Munk model, the reflection value of the dyed textile was transformed to the K/S value by Eq. (2), and the concentration value was estimated by Eq. (11). In the Allen–Goldfinger model, as mentioned above and with an inverse method, the concentration value was calculated from the reflection value of the dyed textile with the dye absorption in the fiber instead of that in the solution. The obtained values of concentration were compared with the actual ones as follows. Two Acid dyes, namely Green 25 and Yellow 25 were selected. Three dyed specimens with specified concentrations at different zones were chosen. The error values of concentration estimation at $\lambda_{\max }$ were calculated and presented in Tables 3 and 4. A comparison between Tables 3 and 4 at low concentrations indicates that for Acid Green 25 at $\lambda_{\max }$, the estimation accuracy is high by the Geometry model, but the result changes for Acid Yellow 25. It is observed that with increasing the concentration, the estimated error value from both models increases. It is due to the high values of concentration estimated by the unit k/s value which originates from the linearity of the reflectance function against the concentration (Fig. 6). As a result, while the outputs of both models at the low concentration values are within the acceptable ranges, the K-M model provided better results in comparison to the Geometry model.

**Table 3:** The error values of concentration estimation by the Geometry and the K-M models at different zones for Acid Green 25 at $\lambda_{\max }$.

Concentration range	Actual concentration (%)	Estimated concentration by geometry model (%)	Error of geometry model (%)	Estimated concentration by K–M model (%)	Error of K–M model (%)
Low (linear)	0.8	0.8	0	0.84	5.34
Medium (non-linear)	2	1.7	15	1.47	26.49
High (saturated)	4	2.5	37.5	1.57	60.53

**Table 4:** The error values of concentration estimation by the Geometry and the K-M models at different zones for Acid Yellow 25 at $\lambda_{\max }$.

Concentration range	Actual concentration (%)	Estimated concentration by geometry model (%)	Error of geometry model (%)	Estimated concentration by K–M model (%)	Error of K–M model (%)
Low (linear)	0.8	0.5	37.5	0.808	1
Medium (non-linear)	2	10	400	0.95	52.34
High (saturated)	4	10	150	0.98	75.36

Although some amendments were mentioned to improve Kubelka–Munk's achievements in estimating the concentration, the results revealed the simple form of the K-M model is still of practical importance and interest in research works (Eq. (11)), but it is limited to the low concentration values^30,51,52^. In reference 38 a technique that extends the applicability of this model to the higher concentration ranges is introduced. The results showed that the Geometry model does not lead to better outcomes in comparison to the K–M model, while it suffers from complex calculations. The fiber properties don’t meet required the hypotheses and the dominant principles of the model as well. Subsequently, it could be concluded that the K-M model is still more reliable in estimating the dye concentration in translucent materials such as textile fibers.

## Conclusions

The main goal of this study was to compare the applicability of two reflective models introduced by the Kubelka–Munk and the Allen–Goldfinger in estimating the dye concentration from the reflectance data of the dyeing system. The results showed the common form of the Allen–Goldfinger model which applies the Beer–Lambert absorption coefficient leads to large error values in the prediction of the spectral reflectance factor. Therefore, the improved Geometry model by using the unit k/s instead of the Beer–Lambert absorption coefficient was applied which prepared a better estimation of spectral reflectance. It was observed that in predicting the reflectance factor based on the replacement of the absorption coefficient of the dye in the fiber instead of the solution, for example, for the Acid Green 25, the prediction errors in the range of low, medium, and high concentrations respectively from 37.93 to 8.45, 63.12 to 14.13 and 93.67 to 21.41 decrease. Furthermore, the results showed that the Allen–Goldfinger model even in the modified forms despite the complexity of computations may not lead to better results in comparison with the K–M model. It was also concluded that the results of error analysis depend on the factors such as the applied concentration range and the spectral behaviors of dyes.

## Data Availability

All data generated or analyzed during this study are included in this published article. The data that support the findings of this study are available from [AMIRKABIR university of Technology] but restrictions apply to the availability of these data, which were used under license for the current study, and so are not publicly available. Data are however available from the authors upon reasonable request and with permission of [AMIRKABIR University of Technology].
